# Impact of the increased asylum seeker population on a UK mental health crisis service

**DOI:** 10.1192/bji.2025.6

**Published:** 2025-08

**Authors:** Helen Lashwood, Mark Toynbee, Bradley Hillier, Derek Tracy

**Affiliations:** 1 Foundation Year 2 Doctor, West London NHS Trust, London, UK; 2 Consultant Psychiatrist, West London NHS Trust, London, UK. Email: m.toynbee@nhs.net; 3 Honorary Consultant Forensic Psychiatrist, West London NHS Trust, London, UK; 4 Chief Medical Officer, South London and Maudsley NHS Foundation Trust, London, UK

**Keywords:** Asylum seekers, refugees, mental health, crisis, psychiatry service provision

## Abstract

**Background:**

A large proportion of applicants for asylum in the UK are housed in contingency hotels while awaiting the outcome of their claim. As the prevalence of severe mental illness (SMI) among asylum seekers is estimated to be around 61%, a measurable impact on mental health services local to these hotels might be expected.

**Aims:**

To evaluate the proportion of asylum seekers on the caseload of a crisis mental health team serving an area with a high concentration of contingency hotels, and to briefly explore the care needs of this population.

**Method:**

Retrospective cross-sectional analysis of all referrals to the Hounslow Crisis Assessment and Home Treatment Team (HCAHTT) in London, UK, between 1 April and 30 September 2023.

**Results:**

Of the 718 referrals to HCAHTT, 536 were taken on the caseload, of whom 9 were asylum seekers. No difference was found in the proportion of asylum seekers on the caseload compared with the general population. Asylum seekers were often moved at short notice, over half required an interpreter and all 9 had a history of trauma.

**Conclusions:**

Asylum seekers housed in Hounslow are not over-represented on the HCAHTT caseload compared with the general population. Given that higher rates of SMI in the asylum seeker population are well-established, this result is more likely to be due to systemic factors than to represent an unusually low rate of asylum seekers experiencing SMI. Asylum seekers tended to require interpretation services and have high rates of reported trauma. Continuity of care is affected by the asylum accommodation process. Better active outreach to vulnerable populations to raise awareness of services might be required.

In 2023, the UK received applications for asylum for 84 425 individuals, which was more than double the number applications in 2019.^1,2^ This mirrors a growing population of forcibly displaced people worldwide, which in 2023 grew to over 117 million people – the largest number ever recorded.^3^

For many of these individuals the journey to the UK is fraught.^4^ It is well-documented that exposure to trauma such as this increases an individual's vulnerability to developing a range of psychiatric disorders; indeed, the prevalence of severe mental illness (SMI) in the UK asylum seeker population has been estimated to be around 61%,^5^ with the World Health Organization predicting that even this is likely to be an underestimate due to under-reporting.^6^ Predisposing factors especially pertinent to this population include psychological trauma, separation from family support networks and the unsettling nature of both impermanent accommodation and undecided asylum applications. A study performed in the North of England found that mental ill health was widely considered the greatest issue for the asylum seeker community, with support groups expressing concern over suicides and fatal interpersonal violence.^7^

## Asylum seekers in Hounslow

In March 2023, 69% of asylum seekers in the London Borough of Hounslow were housed in contingency hotels while awaiting the outcome of their asylum claim.^8^ In the same month, Hounslow was the borough with the second highest number of asylum seekers accommodated in hotels in London.^8^

Mental healthcare, as part of secondary care services, is available free of charge in the UK for asylum seekers with an active application or appeal.^9^ Hotels housing asylum seekers in Hounslow fall under the catchment area of the Hounslow Crisis Assessment and Home Treatment Team (HCAHTT).^10^ This is a community-based service which provides support to adults, aged 18 and over, in mental health crisis with intensive home treatment. Referrals are received via a number of sources – including liaison psychiatry services from local acute hospitals or the local Single Point of Access (SPA) telephone service, which itself can take referrals from a wide variety of sources, such as general practitioners, the police and members of the public, including staff in the aforementioned hotels. While on the HCAHTT caseload, patients may access input from a wide range of professionals, such as mental health nurses, occupational therapists, psychologists, doctors, support workers and other health and social care practitioners.

The prevalence of SMI in people aged 15–74 in England is reported to be between 0.7 and 0.9%.^11^ However, as mentioned above, the prevalence of SMI in the asylum seeker population is reported to be as high as 61%.^5^ A similar trend is seen for common mental disorders: compared with the general population, asylum seekers have nearly double the rates of anxiety and are almost ten times more likely to experience depression. In adults, post-traumatic stress disorder has a prevalence of 31% in asylum seekers, compared with 3.7% among men and 5.1% among women in the general population in England.^12,13^

Therefore, as asylum seekers comprised 0.8% of Hounslow's population in March 2023,^8^ if there is equality of access we expect the proportion of asylum seekers on the HCAHTT caseload to be greater than 0.8%.

Given the high reported prevalence of SMI among asylum seekers, as well as the rapidly increasing numbers of asylum seekers in the UK, it might be expected that this population would produce demonstrable increase in demand on National Health Service (NHS) mental health services local to contingency hotels where they are temporarily housed.

Moreover, further complexities such as specific trauma-related presentations, language barriers, accommodation issues and potentially adverse experiences of authority, including health services, might be expected in this population.

## The present study

To inform service provision regarding this potential increase in demand, we performed a retrospective evaluation examining referrals to HCAHTT over a 6-month period.

We aimed to explore whether asylum seekers housed locally were more likely to be on the caseload of HCAHTT than the general Hounslow population.

As a secondary aim, possible barriers to access for asylum seekers were identified, as these have potential important implications for patient care and service design. A more in-depth analysis of the specific needs of various groups within the asylum seeker population was not possible owing to the small number of asylum seekers that were identified on the HCAHTT caseload.

Specifically, we aimed to:

- identify all referrals of asylum seekers to HCAHTT;
- identify sources of referrals for asylum seekers on the HCAHTT caseload;
- identify whether any referred asylum seekers were not taken on the caseload to be supported by HCAHTT;
- establish the proportion of asylum seekers on the HCAHTT caseload;
- compare the above with the proportion of asylum seekers in Hounslow's population;
- identify ongoing care destinations for asylum seekers on discharge from HCAHTT;
- identify the proportion of asylum seekers who require an interpreter;
- identify the proportion of asylum seekers who have trauma documented in their notes, as well as whether this occurred pre- or post-migration or during the migratory journey.

## Method

### Data collection period

All referrals to HCAHTT from 1 April 2023 to 30 September 2023 were examined. The start date of 1 April 2023 was chosen as this is when Hounslow Local Authority reported that the experiences of asylum seekers housed in hotels in the borough saw a significant deterioration.^14^ This was multifactorial, but notably included a change in policy to allow multiple strangers to share one hotel room.^14^ The end date of 30 September enabled direct comparison with data collected nationally, which are reported by borough and recorded per quarter, and also reflected a period of stability in the number of asylum seekers who were in receipt of support from the local authority.^15^ Multiple referrals for the same individual within this period were counted separately.

### Data collection protocol

All referrals were screened to identify the registered address of each patient, identifying those housed in Hounslow contingency hotels.

The length of time each referral was open was calculated, from date of referral to date of discharge. If the duration was less than 1 day the referral was excluded, as this indicated that the referred individual was not supported by HCAHTT.

Further exclusion criteria were then applied, identifying any other referrals that were not taken on because:

- initial assessment concluded there was no role for HCAHTT;
- the referral was directed to a different service as the individual was out of area.

For every individual identified as housed in a contingency hotel in the excluded group, the reason that they were not taken on to the caseload was documented.

For every individual in the included group, which represented those taken on to the caseload, a second check was performed by searching the electronic patient record for the terms ‘asylum’ and ‘refuge’ to identify any asylum seekers who may have been resident in a contingency hotel at the time of HCAHTT input but whose registered address had subsequently changed.

Following this, the proportion of asylum seekers on the caseload was compared with data on the proportion of asylum seekers in Hounslow's population. The likelihood of any Hounslow resident being supported by HCAHTT is discrete and well-modelled by a binomial distribution (either ‘supported’ or ‘not supported’). Therefore, whether the number of asylum seekers in Hounslow's population was proportionate to the number supported by HCAHTT could be tested using a hypothesis test. The hypothesis under test was that asylum seekers were over-represented in the caseload.

Finally, electronic patient records of the asylum seekers housed in hotels in the included group were searched to identify:

- which service had made the referral to HCAHTT;
- which, if any, ongoing health services were involved in care on discharge from HCAHTT;
- any mention of the terms ‘language’ and ‘translat*’ to identify information regarding interpreter requirements;
- mention of the term ‘trauma’, to identify whether trauma was discussed and, if documented, whether the trauma occurred pre- or post-migration or was related to their journey.

## Results

### The number of asylum seekers referred

A total of 718 referrals were received by HCAHTT in the review period. Eleven of those referrals were for asylum seekers housed in a contingency hotel. Two individuals were excluded from the analysis as their referrals were discharged within 1 day and were therefore judged not to have received input from HCAHTT. One individual was immediately transferred to a neighbouring borough's CAHTT as this team was able to offer a Crisis House service. The other referral was for a gatekeeping assessment, where the individual was admitted directly to a psychiatric ward.

### The number of asylum seekers accepted on to the HCAHTT caseload

Of the 718 referrals, 536 were accepted on to the caseload, of whom 9 were legally classed as asylum seekers with ongoing applications. The location (anonymised hotels A–D) of asylum seekers who were accepted on to the HCAHTT caseload are summarised in Table 1.

**Table 1:** Location of asylum seekers (*n* = 9) on the caseload at point of referral to the Hounslow Crisis Assessment and Home Treatment Team during the evaluation period

Location	Asylum seekers, *n*
Asylum Hotel A	2
Asylum Hotel B	5
Asylum Hotel C	1
Asylum Hotel D	1

One individual was identified who had recently applied for asylum but had been resident in private housing in the UK for a number of years, and so was not included in the asylum seeker data.

### Comparison of proportion of asylum seekers on caseload with latest data on proportion of asylum seekers in Hounslow

In March 2023, asylum seekers made up 0.8% of the resident population of Hounslow,^8^ meaning that an approximation of the probability of a person in Hounslow being an asylum seeker is *P* = 0.008.

Given that 9 of 536 individuals supported by HCAHTT were asylum seekers (1.68%), an approximation of the probability of a person on the HCAHTT caseload being an asylum seeker is *P* = 0.0168.

Therefore, as 0.0168 > 0.008, the hypothesis under test was that asylum seekers were over-represented in the caseload. As there were 9 asylum seekers on the HCAHTT caseload, it was tested whether 𝑃(𝑋 ≥ 9) is significant for this distribution with *n* = 536 and *P* = 0.008.

Using standard binomial distribution calculation, we can see that 𝑃(𝑋 ≥ 9) = 0.03065, and therefore this result is not statistically significant at the *P* < 0.01 level; asylum seekers have not been found to make up a higher proportion of the caseload than other members of the general population.

### Source of referral for asylum seekers accepted by HCAHTT

The source of referral for asylum seekers on the caseload showed a similar pattern to that of the referrals for patients in the general population (Table 2).

**Table 2:** Source of referral to the Hounslow Crisis Assessment and Home Treatment Team for asylum seekers and the general population, April–September 2023

Source of referral	General population referrals, %	Asylum seeker referrals, %
Liaison psychiatry service	49	67
In-patient	19	11
Community mental health team	14	0
Single Point of Access service	11	11
Out of area	7	11

### Continuity of care after discharge from HCAHTT

Regarding ongoing care provision, data were collected regarding transfer of care (TOC) for each asylum seeker, summarised in Table 3.

**Table 3:** Ongoing care provision for patients classed as asylum seekers (*n* = 9)

Ongoing care provision	Asylum seekers, *n*
General practitioner (GP)	4
Community mental health team	2
Home treatment team elsewhere	1
Unable to transfer care successfully as patient not registered with GP	2

For six individuals, TOC to an NHS care provider was possible: for four individuals this was to their general practitioner, and for two it was to the community mental health team.

Three individuals were moved out of area by the Home Office while HCAHTT input was ongoing. For one, TOC to another home treatment team was possible. However, for the other two individuals HCAHTT was not able to identify where they had been moved to and so TOC was not possible.

### Interpretation requirements for asylum seekers

For five of the nine asylum seekers, an interpreter was required to facilitate assessments. Their language requirements are described in Table 4.

**Table 4:** Summary of language requirements of asylum seekers identified while under the Hounslow Crisis Assessment and Home Treatment Team

Asylum seeker	Language requirements
A	‘fairly able to communicate in English but prefers [ … ] interpreter’
B	‘spoke fluent [ … ] English’
C	‘happy to converse in English and does not want an interpreter’
D	‘limited English [ … ] Assessment conducted via Silent Sounds’
E	‘Assessment performed via Language Line since no in-person interpreter present’; ‘needs specialist help with Arabic language capability’
F	‘interpreting over the phone [ … ] facilitated by an interpreter’
G	Fluent English
H	No language requirements identified
I	‘language barrier [ … ] native language of Farsi’

### Prevalence of trauma in asylum seekers on HCAHTT caseload

**Fig. 1:**
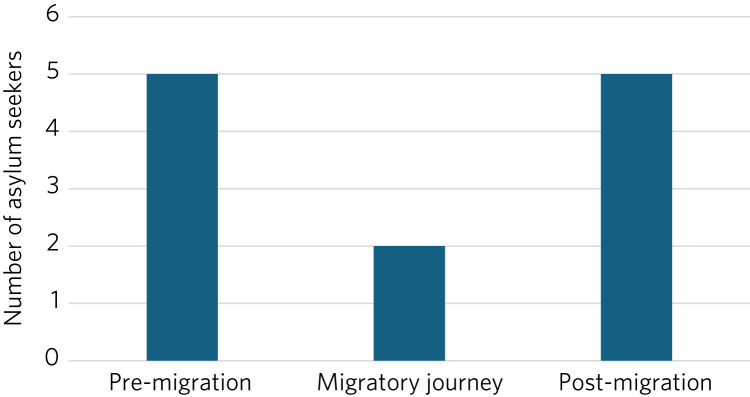
Distribution of timing of reported trauma by asylum seekers (*n* = 9). Each individual could have experienced trauma at more than one time point.

A search of the electronic patient records for references to trauma revealed that all nine asylum seekers disclosed they had experienced this. The distribution of when in their migratory journey this was reported (which could be at multiple points for each individual) is shown in Fig. 1.

## Discussion

The evaluation found that all 11 asylum seekers referred to the Hounslow Crisis Assessment and Home Treatment Team (HCAHTT) between April and September 2023 were either accepted or referred to other secondary care services. Nine of the eleven were taken on to the HCAHTT caseload to receive support, representing 1.68% of the total caseload. Against prediction, no significant difference was found between asylum seekers and the general population regarding likelihood to be supported by HCAHTT. Given the higher prevalence of both trauma and SMI in the asylum seeker population reported in the literature, it is possible that acute mental health requirements in the Hounslow asylum seeker population were under-recognised or under-reported upstream of referral. It is therefore important to consider what barriers to access may exist for this population when seeking mental healthcare.

### Factors potentially impairing service accessibility

Owing to the nature of HCAHTT's service (intensive home support, within 4 h if necessary, at no financial cost), commonly cited barriers to accessing care, such as long waiting lists, costly travel and affordability, were not directly applicable.^16^ Despite this, there are a number of factors to consider that may impair HCAHTT's accessibility for this population, including awareness, cultural attitudes to mental illness and treatments, and concerns about confidentiality, trust and how engagement might affect any asylum claim, as well as service-based barriers such as availability of culturally and linguistically informed information.

#### Awareness

Awareness of crisis mental health services may be one barrier. Referrals to HCAHTT mainly come from other mental health services rather than the local Single Point of Access telephone service available to the general public, including asylum seekers. Accessing this service requires knowledge of its existence and ability to call and engage with them, or registration with a general practitioner.

#### Cultural attitudes

Where knowledge of services exists, cultural attitudes towards mental illness may still be a barrier. One cultural difference that may prevent asylum seekers accessing services when in crisis is a difference in help-seeking behaviour. Given the heterogeneity of cultures that comprise the UK asylum seeker population it is outside the scope of this evaluation to comment on the full variety and nuance of differing help-seeking behaviours. However, it has been shown that individuals in both Kurdish and Somali communities may prefer to seek help from a community figure or traditional/faith healer prior to seeking help from NHS services,^17,18^ which would delay access to NHS services. Consequently, improving links with community organisations and leaders through engagement and educational activities could be an important way to ensure earlier access to services.

Stigma may also be operative, with one systematic review identifying several studies in which asylum seekers expressed concern they may be stigmatised by their communities for seeking mental health support.^16^ This again emphasises the need for engagement and information about the nature of mental health conditions and their treatability.

#### Service-based barriers

Service acceptability may be a barrier.^19^ It has been shown that where healthcare staff do not share a cultural background with an asylum seeker, trust can be impaired, and complex intersectional factors such as gender, age and religion might have a further impact on this.^20^ Individuals might also have concerns about how identification of a mental illness might affect any asylum claim. Furthermore, past traumatic experiences might create hesitation regarding places and figures of authority. All asylum seekers who were referred to HCAHTT were offered treatment in secondary care; however, this hesitation may have been a factor upstream of referral – for instance, those who did not expect mental health services to demonstrate sensitivity to their needs may not have sought help.

One method to make mental healthcare services more acceptable may be to include peer support workers, which has been shown to improve service accessibility for asylum seekers.^21^

#### Ongoing care needs

When treating individuals resident in contingency hotels there is always potential that the individual will be moved by the Home Office to alternative accommodation outside the local area, as occurred with three of the nine individuals in this evaluation. When this happens, the new address is not routinely shared by the Home Office with either HCAHTT or the hotels they were previously resident in. This can lead to an abrupt end to the interventions being provided by the service and a successful transfer of care cannot be performed. Given the language barriers identified in this evaluation, it may be that some of these individuals felt unable to telephone HCAHTT to inform them of a change of address, which may indicate the need for this to be specifically planned for at the start of treatment.

Consequently, lack of continuity in care contributes to the instability this group experiences, increasing the likelihood of duplicated initial assessments, having to repeat (often traumatic) histories to a new team who are unable to view previous records.

#### Interpreter requirements

Additional care needs arise from the high prevalence of language barriers in this population. Language barriers are not exclusive to asylum seekers but were common in our group (Table 4). HCAHTT staff rely on professional interpretation services, mainly via teleconference; although this is preferable to using informal interpreters such as family members,^22^ professional interpretation services also have limitations. For example, any nuance implied in the phrasing used by both the healthcare professional and the patient may be lost in translation because of differing cultural backgrounds despite a shared language as so much of the cultural and linguistic content of language does not directly translate. Additionally, when conducted over telephone, the absence of non-verbal cues may further predispose to transcultural misunderstandings.

The breadth of factors involved in culturally sensitive care is outside the scope of this evaluation, but given the frequency of language problems it is relevant to note briefly the importance of awareness of ‘cultural idioms of distress’. Each culture has its own idioms that carry significance for members of that culture. For instance, in Western European cultures one common cultural idiom of distress centres on the concept of back pain. Phrases such as ‘a weight on my shoulders’, ‘a pain in the neck’ and ‘more than I can bear’ all refer to psychological distress.^23^ Translated word for word into another language, these metaphors lose some of their significance.

Furthermore, these idioms can transcend metaphor, with psychological distress manifesting as physical pain.^24^ The intersection between psychological and physical pain has been shown to be of great significance, with physical pain in those who have comorbid depression having been shown to be more intense and protracted than in those who do not have depression.^16,24^ If these cultural idioms are not recognised, the depth of complexity and meaning that is so critical to a phenomenological understanding of an individual's experience and subsequent formulation of a psychiatric diagnosis is lost.

#### Trauma

The findings of high recorded rates of traumatic experiences in the nine asylum seekers identified are consistent with similar studies performed on a much wider scale.^25^ Such a high degree of trauma predisposes this population not only to post-traumatic stress disorder but also to significant complexity in which multiple clusters of symptoms are present to a clinically significant level.^26^ Such situations are likely to require a higher intensity of intervention.

Furthermore, those who have experienced trauma are less likely to seek support via formal pathways and are generally more likely to self-medicate using drugs or alcohol, adding further complexity to their presentation, including additional barriers in accessing services.^27^

### Strengths and limitations

The strengths of this evaluation include: (a) a geographical area with the second highest number of asylum seekers housed in hotels in London; (b) a well-defined population with clear inclusion and exclusion criteria; (c) comprehensive review of all referrals over a 6-month period; (d) thorough review of all identified asylum seekers’ care needs relevant to a crisis mental health service.

The limitations of this evaluation include: (a) that it is a retrospective data review; (b) the unexpectedly small number of asylum seekers in the study population; (c) uncertain generalisability to other settings; (d) the statistical test used involves assumption that an asylum seeker referred to the Single Point of Access service or liaison psychiatry services is equally likely to be referred on to HCAHTT if they meet the referral criteria as any other resident of Hounslow.

### Wider context

This evaluation builds on previous work on provision of mental healthcare for asylum seeker populations in the UK. For instance, a study in the North of England that investigated a range of asylum seeker support services identified similar issues to those identified in this evaluation. It recognised inadequate signposting to services, poor communication between services and that bespoke trauma-informed services with sufficient provision for language interpretation were few and far between. As far as we are aware, this is the first study specifically to assess crisis team accessibility to asylum seekers in the UK.^7^

### Future directions

#### Interventions to increase awareness of available mental health services

Further work to raise awareness and understanding, both among staff and the resident population of contingency hotels, of what mental health support is available may increase the numbers of asylum seekers who contact services. Seeking the views of asylum seekers and representatives of the various communities represented in the hotel population in a culturally sensitive way might help identify potential needs and barriers to access, and co-developed written literature in multiple languages can raise awareness of what services are available and how to contact a Single Point of Access service. The information could be provided to contingency hotels, who already routinely advertise written information that promotes local initiatives to residents. Actively seeking to intervene earlier in this way may improve outcomes for asylum seeker mental health in the longer term, subsequently promoting social inclusion for those who are granted settled status.

Furthermore, as most referrals came from liaison psychiatry services, it is reasonable to assume that many asylum seekers will present to the emergency department when seeking healthcare. As such, this written information could be made available in a local emergency department to promote awareness amongst those who may be presenting with physical health concerns and therefore not be otherwise referred to liaison psychiatry services.

#### Train and equip service providers

Currently, there is a no mandatory training that seeks to equip local healthcare providers to provide a culturally sensitive approach to supporting asylum seekers. Given the heterogeneity of the asylum seeker population, no such training could exhaustively encompass the nuances of working with such a diverse population. However, even if training seeks only to provide an awareness of various cultural explanations of distress, this may promote more compassionate care as staff would be better equipped to recognise and adapt to differences in worldview. Furthermore, it has been shown that training in refugee and migrant-specific healthcare maps to five of the General Medical Council's recommended outcomes for undergraduate medical students.^28^

Following the findings of this evaluation, local training to enhance understanding of the issues most pertinent to the asylum seeker population has been run within HCAHTT. As part of this, the multidisciplinary team was invited to discuss the findings of the evaluation, seeking to open a conversation about how HCAHTT might best serve individuals in contingency hotels.

Moreover, given that the number of asylum seekers taken on to the caseload is below what is expected, it is likely to be beneficial to initiate training for potential referrers, particularly local liaison psychiatry services. This would aim to promote consideration of barriers to care for asylum seekers, such as those set out above.

#### Interpreter services

As highlighted by this evaluation, access to timely interpretation services is necessary both for those seeking support to feel able to do so, as well as for healthcare providers to feel able to perform a comprehensive assessment. However, it is possible that, particularly in smaller immigrant communities, some individuals might have concerns about being known to an interpreter.

Additionally, easy access to interpretation is likely to improve accessibility of services by phone – for instance, enabling those who do not speak English to be able to inform services when they are moved to a new address.

## Data Availability

Anonymised data that support the findings of this study are available on request from the corresponding author, M.T., subject to NHS confidentiality rules. The data used were routinely collected, confidential NHS data.
